# Supplementary material for: The effects of genital myiasis on the diversity of the vaginal microbiota in female Bactrian camels
Source: BMC Vet Res. 2022 Mar 5;18:87. doi: 10.1186/s12917-022-03189-5 (PMC8897907; doi:10.1186/s12917-022-03189-5)
Supplement: Supplementary file 5 — Additional file 5. [file 12917_2022_3189_MOESM5_ESM.zip › MPL201709200_16s_yy/Treat1/css/js_css/pdfmake-master/examples/pdfs/tables.pdf]

Tables

Official documentation is in progress, this document is just a glimpse of what is possible with pdfmake and its layout engine.

A simple table (no headers, no width specified, no spans, no styling)

The following table has nothing more than a body array

Column 1	Column 2	Column 3
One value goes here	Another one here	OK?

A simple table with nested elements

It is of course possible to nest any other type of nodes available in pdfmake inside table cells

<table>
<tr><td>Column 1</td><td>Column 2</td><td>Column 3</td></tr>
<tr><td>Let's try an unordered list
item 1
item 2</td><td>or a nested table<table><tr><td>Col1</td><td>Col2</td><td>Col3</td></tr><tr><td>1</td><td>2</td><td>3</td></tr><tr><td>1</td><td>2</td><td>3</td></tr></table></td><td>Inlines can be styled
easily as everywhere else</td></tr>
</table>

Defining column widths

Tables support the same width definitions as standard columns:

- **auto**
- **star**
- **fixed value**

width=100	star-sized	width=200	star-sized
fixed-width cells have exactly the specified width	*nothing interesting here*	*nothing interesting here*	*nothing interesting here*

Headers

You can declare how many rows should be treated as a header. Headers are automatically repeated on the following pages

It is also possible to set keepWithHeaderRows to make sure there will be no page-break between the header and these rows. Take a look at the document-definition and play with it. If you set it to one, the following table will automatically start on the next page, since there's not enough space for the first row to be rendered here

**Header 1**	**Header 2**	**Header 3**
Lorem ipsum dolor sit amet, consectetur adipisicing elit, sed do eiusmod tempor	Lorem ipsum dolor sit amet, consectetur adipisicing elit, sed do eiusmod tempor	Lorem ipsum dolor sit amet, consectetur adipisicing elit, sed do eiusmod tempor

Header 1	Header 2	Header 3
incididunt ut labore et dolore magna aliqua. Ut enim ad minim veniam, quis nostrud exercitation ullamco laboris nisi ut aliquip ex ea commodo consequat. Duis aute irure dolor in reprehenderit in voluptate velit esse cillum dolore eu fugiat nulla pariatur. Excepteur sint occaecat cupidatat non proident, sunt in culpa qui officia deserunt mollit anim id est laborum.	incididunt ut labore et dolore magna aliqua. Ut enim ad minim veniam, quis nostrud exercitation ullamco laboris nisi ut aliquip ex ea commodo consequat. Duis aute irure dolor in reprehenderit in voluptate velit esse cillum dolore eu fugiat nulla pariatur. Excepteur sint occaecat cupidatat non proident, sunt in culpa qui officia deserunt mollit anim id est laborum.	incididunt ut labore et dolore magna aliqua. Ut enim ad minim veniam, quis nostrud exercitation ullamco laboris nisi ut aliquip ex ea commodo consequat. Duis aute irure dolor in reprehenderit in voluptate velit esse cillum dolore eu fugiat nulla pariatur. Excepteur sint occaecat cupidatat non proident, sunt in culpa qui officia deserunt mollit anim id est laborum.

Column/row spans

Each cell-element can set a rowSpan or colSpan

Header with Colspan = 2		Header 3
**Header 1**	**Header 2**	**Header 3**
Sample value 1	Sample value 2	Sample value 3
rowSpan set to 3<br>Lorem ipsum dolor sit amet, consectetur adipisicing elit, sed do eiusmod tempor	Sample value 2	Sample value 3
	Sample value 2	Sample value 3
	Sample value 2	Sample value 3
Sample value 1	Both:<br>rowSpan and colSpan<br>can be defined at the same time	
Sample value 1		

Styling tables

You can provide a custom styler for the table. Currently it supports:

- line widths
- line colors
- cell paddings

with more options coming soon...

pdfmake currently has a few predefined styles (see them on the next page)

noBorders:

Header 1	Header 2	Header 3
Sample value 1	Sample value 2	Sample value 3
Sample value 1	Sample value 2	Sample value 3
Sample value 1	Sample value 2	Sample value 3
Sample value 1	Sample value 2	Sample value 3
Sample value 1	Sample value 2	Sample value 3

headerLineOnly:

Header 1	Header 2	Header 3
Sample value 1	Sample value 2	Sample value 3
Sample value 1	Sample value 2	Sample value 3
Sample value 1	Sample value 2	Sample value 3
Sample value 1	Sample value 2	Sample value 3
Sample value 1	Sample value 2	Sample value 3

lightHorizontalLines:

Header 1	Header 2	Header 3
Sample value 1	Sample value 2	Sample value 3
Sample value 1	Sample value 2	Sample value 3
Sample value 1	Sample value 2	Sample value 3
Sample value 1	Sample value 2	Sample value 3
Sample value 1	Sample value 2	Sample value 3

but you can provide a custom styler as well

Header 1	Header 2	Header 3
Sample value 1	Sample value 2	Sample value 3
Sample value 1	Sample value 2	Sample value 3
Sample value 1	Sample value 2	Sample value 3
Sample value 1	Sample value 2	Sample value 3
Sample value 1	Sample value 2	Sample value 3
